# Fellow cows and conflicting farmers: Public perceptions of dairy farming uncovered through frame analysis

**DOI:** 10.3389/fvets.2022.995240

**Published:** 2022-11-17

**Authors:** Amy Jackson, Martin J. Green, Jasmeet Kaler

**Affiliations:** Ruminant Population Health, The School of Veterinary Medicine and Science, University of Nottingham, Nottingham, United Kingdom

**Keywords:** frame analysis, animal welfare, dairy cow, dairy farmer, reflexive thematic analysis

## Abstract

Divergence in opinion over how farm animals should be cared for is creating a disconnect between livestock farming and the public that risks a loss of “social license” to farm. One proposed solution for the dairy farming community is to engage more constructively with the public to develop a shared vision of the industry's future; however, farmers and veterinarians remain reluctant to validate public opinions on farm animal care, in particular, often viewing them as naïve or impractical. Understanding the interpretive frames through which people make sense of dairy farming could help the dairy farming community engage more constructively with public opinion, thereby reducing conflict and providing opportunities to change communication or practice. Hence, frame analysis was conducted on transcripts of 60 face-to-face interviews with members of the UK public, first defining frames using reflexive thematic analysis, then considering the effect of these frames on those holding them. The results showed that dairy farming was mainly characterized by two entities: the cow and the farmer. Three frames were developed for the cow: she was perceived as i) enduring, which induced a sense of moral responsibility for her well-being among participants; ii) a fellow or companion, which led to feelings of a shared or parallel life with her; and iii) a force of nature, where the cow's connection with the natural world and “otherness” was appreciated, or even longed for. These connections were unexpectedly widespread within the sample, with many participants simultaneously holding two or even three frames. The farmer was seen through two frames: i) traditional; or ii) modernizing, but both frames had positive and negative narratives depending on the perceived care of the cow, causing confusion or even conflict about the care the farmer actually delivered. These findings provide new insights into the interpretive lenses through which the public makes sense of the dairy cow and her care, not least the bond the public themselves feel with the animal. They offer fresh opportunities for the dairy industry to improve engagement through more reflexive communication or modification of farming practices to better fit societal expectations about dairy cow welfare.

## Introduction

It is almost 60 years since Ruth Harrison first brought concerns about increasingly intensive farming methods and their impact on the farm animals involved to public attention through her seminal work *Animal Machines* (1). Despite this, the farming industry and external audiences continue to diverge over how farm animals should be kept (2), with public dissatisfaction about current methods expressed through a range of vehicles including survey results (3, 4), online campaigns (5, 6), government policy (7, 8), and product development (9, 10). However, there is evidence that farmers and veterinarians dismiss such concerns on grounds of the public being uninformed about farming (11–14), unaware of the realities of livestock production (15, 16), influenced by animal rights advocates (11, 12, 17), prone to anthropomorphism (11), or naïve about the economic impacts of changing practices (18). As well as this, farmers and farm industry representatives have expressed frustration across a variety of public media about “being told how to farm” by those they believe lack knowledge of the industry or its technicalities (19–21).

Yet there are consequences to overlooking public opinion. Public disquiet about the sustainability of livestock farming has the potential to impact its “social license” to operate, reducing the (often tacit) permission that communities or wider society grant for the utilization of land and other resources (22). Recently, concerns over the industry's impact on the environment and animal welfare, in particular, have been expressed in both consumer and citizen surveys (23, 24), given as key reasons for conversion to veganism (25, 26), and explained as leading motivators for engagement in activism (27).

In their examination of how the dairy industry in particular should address this disconnect with public views, Weary and Keyserlingk (28) have concluded that if dairy farms are to survive, the industry needs to work constructively with external stakeholders to develop a shared vision of its future. The challenge, therefore, is to encourage a desire for cooperation and mutual understanding within the dairy industry—despite an apparent reluctance to recognize the validity of societal concerns, or, indeed, change to address them (16).

An approach to resolving this divide is suggested by Shmueli et al. (29), who report that a better knowledge of the interpretive frames through which people characterize others, and how they have been constructed, can improve empathetic reflection and reduce conflict. This is logical when considering that frames have variously been described as schemas of interpretation which allow their users to identify and label information (30), cognitive structures that fill gaps in perception (31), and “data structures” that present stereotyped situations to make sense of the new (32). Despite this, to our knowledge, the frames, or “lenses,” through which the public interprets farming have not previously been examined. Understanding how dairy farming is perceived, and the diversity of interpretive frames employed to form that perception, has the potential to explain how the public views practices, why they express certain preferences, and what they hope to achieve by doing so. Hence, in this study, we adopt the novel use of frame analysis to uncover the interpretive lenses through which a sample of the public views dairy farming. We then discuss what knowledge of these frames might mean for the dairy industry, and whether enhancing this understanding may cultivate more empathy among farmers and veterinarians for wider societal views, help to find common ground with the public—and even prompt changes to communication or practice.

## Methods

### Approach

Framing is often used deliberately to convey meaning or position an issue in a particular light, making it more important how something is communicated rather than what is communicated [e.g., (14, 33). However, frames have also been examined reflectively to understand how people utilize former interactions, experiences, memories, feelings, associations, and other fragments of information to either make sense of a situation or stimulus (“cognitive frames”) or to guide a context-specific interaction (“interactional frames”) (34, 35). In the case of the former, analyzing the frames people “hold,” literally as frames of reference, can help our understanding of how they make sense of societal issues, for example: by what means do healthcare professionals justify intervention or non-intervention in domestic violence (36); the ways in which young people value public spaces (37); and how consumers rationalize the acceptability—or not—of eating meat (38). In terms of livestock farming, the study of cognitive frames has been successfully applied to various communications-related challenges, such as how the term “positive welfare” is construed by different audiences (39), the ways in which veterinarians perceive the problem of poor biosecurity (40), and whether an appreciation of each other's perspectives can help pig farmers and the public find common ground on animal husbandry (16).

### Data collection

Qualitative research methods are commonly used to analyze subjective human experiences which give rise to phenomena such as framing (41, 42). Variation within the data being analyzed improves the outcome of frame analysis because it allows the identification of contrasts and similarities within a diverse sample as they discuss the same themes (43). In this instance, face-to-face interviews were used as it was believed they would elicit a broader and richer range of insights from participants rather than the more consensual outputs that can sometimes be generated by focus groups (43, 44).

A semi-structured interview guide was developed to aid data collection (44, 45), allowing topics to be approached in an intuitive order—yet helping the interviewer keep track if interviewees addressed topics out of order. It also provided interviewees with the opportunity to ask questions, interpret questions in their own way, and introduce novel components to uncover a wider range of meanings (41). To address our research objective, the interview script asked participants to think of a dairy farm and then describe the image that came to mind and where it had come from. Prompts were used to add clarity to what the entities described looked like or were doing. To obtain responses based on participants' frames of reference, these questions were positioned near the start of the guide to create the principal data analyzed. However, the interview guide also sought participant views on a wider range of aspects related to dairy farming, intended for analysis within other studies. It was anticipated that participants might access frames to help them answer these other questions, and if this occurred, these data would be included for analysis as well. The interview guide was piloted with five colleagues to test and modify the interview script, with most adjustments being made to the running order and prompts. The final and complete interview guide is available as Supplementary material.

### Recruitment

Before commencing the recruitment of participants, ethical approval was received from the University of Nottingham School of Veterinary Medicine and Science's Research Ethics Committee (no. 1860 160930). Market research company Made In Surveys (MIS, Lille, France; https://en.misgroup.io) identified potential interviewees using purposive sampling (46) from over 2,000 participants who took part in Jackson et al. (47). The aim of drawing from this larger sample was to have a broad representation of the six citizen groups identified by Jackson et al. (47) within the qualitative sample. Additionally, because of the importance of variation within data used for frame analysis (43), other aspects of diversity were sought. Primarily, as experience of rural living was known to significantly impact preferences for farm animal welfare (47–53), the aim was that approximately half of the participants should come from rural areas or have rural living experience. An even split on gender, and a range of age and geographical location was also requested, due to the impact of gender and age on preferences (47, 50, 53) and variations in personality and political, economic, social, and health indicators across UK regions (54).

The “information power” approach was adopted to estimate the required sample size (55). Although face-to-face interviews would usually provide a high degree of information power, several factors reduced this, including the breadth of the research objective, the specificity needed for a purposive sampling approach, the open-ended nature of the questions, and the use of inductive reflexive thematic analysis (56) to conduct frame analysis. Thus, it was indicated a relatively large sample would be needed. To establish parameters for the size of a qualitative interview sample, 50–60 interviews have been proposed as normal in large qualitative samples (57), but more than 50 can also present a challenge with analysis (58, 59). It was nonetheless decided to aim to collect data from 60 interviews but monitor the breadth of data and diversity of participants as the interviews progressed in case numbers could be adjusted. At the conclusion, all 60 interviews were executed because many involving rurally based participants took place later in the schedule, and there were challenges with obtaining sufficient interviews from people representing one of the six citizen groups in particular.

### Conducting interviews

All participants received an information sheet once they provisionally agreed to be interviewed, detailing measures to protect anonymity and data, and compliance with General Data Protection Regulation 2016/679; these were sent out in advance, with participants needing to have provided written consent to these terms before the actual interview took place. It was confirmed that each participant would be remunerated after the interview and a post-interview survey. The post-interview survey collecting sociodemographic and experiential data was conducted on a tablet using SurveyMonkey (Momentive Inc., San Mateo, California; www.momentive.ai).

Sixty semi-structured face-to-face interviews with members of the public across the UK were conducted from November 2019 to February 2020. The same author (AJ) undertook all interviews on her own; they lasted between 25 and 90 min and averaged an hour. Participants were interviewed once, and interviews were transcribed from the audio recordings using the intelligent verbatim method to optimize readability and meaning (60), and checked against audio recordings and field notes during analysis. Inaudible comments that could not be rectified were excluded. Audio recordings and transcripts were anonymized, with identifying details password-protected and stored securely for data protection purposes.

### Positionality

It is recognized that the interviewer and those undertaking qualitative research and analysis are themselves research instruments, and their positionality affects both data gathering and interpretation (61, 62). While the author who conducted the interviews (AJ) is not a farmer and does not come from a farming background, she has practical and theoretical understanding as well as experience in farming. She was also cognizant of the potential for a social desirability bias effect (63, 64) to influence responses within face-to-face interviews. AJ, therefore, focused on maintaining outward neutrality during interviews, avoiding responses that may have suggested personal views or background. She was also conscious of the need to interpret interviewee comments about dairy farming using their experiences rather than her own.

### Analysis

The interview transcripts were uploaded into NVivo 12 (QSR International; www.qsrinternational.com) to assist with coding and analysis. Upon review of the transcripts, additional data from the wider interview were included for analysis as anticipated; this was where there were signs that participants were expressing a perception of dairy farming based on cognitive framing. This was indicated, for example, by the expression of information as statements or facts, rather than as guesses or assumptions. Responses during which the participant hypothesized or “thought aloud,” forming their response as they spoke based on the interaction with the interviewer, were excluded as these were judged to be interactional rather than cognitive framing.

While frame analysis encompasses the wider examination of data for the use of frames, it does not proscribe any specific technique. Indeed, Goffman (30) describes no definitive steps. However, there are commonly two stages employed in frame analysis: identifying the frames used, and then the effects of these frames on those holding them (65). Here, our initial identification of the frames employed was undertaken through reflexive thematic analysis of the data (56) using the constant comparative method based on grounded theory (66) and applying the researcher's own experiences and insights to aid interpretation. Initially, words and sections of speech were open-coded into candidate codes which were discussed between authors. The questions asked by the researcher of herself during the analysis were: “What is going on here?” and “How can I explain it?”; these were used repeatedly while coding to retain focus on latent (underlying) rather than semantic (surface) meaning (56), and code proliferation was avoided by reusing existing codes where possible (67). To ensure the reliability of the coding, a sample of transcripts was examined by another researcher (JK). Observations and connections were recorded as the process continued, as was any *in vivo* text which captured a mood or theme. After this first round of coding, codes were arranged several times into different organizing concepts until a narrative was developed that remained coherent despite subsequent coding reviews (41). Several minor themes were identified but as we were seeking to create the key frames through which the participants perceived dairy farming, we discarded those which did not inform this objective. Then at the second stage of frame analysis, the frames were examined to understand what effect they might have on those holding them, and how they might shape the holder's perception of and response to the topic of dairy farming.

## Results

### Participant characteristics

Key aspects relating to the final sample are summarized in the Supplementary material. In short, half of the participants (30/60) were educated to a university degree or post-graduate level; almost half (46–28/60) were professionally or clerically employed, 37% (22/60) did not work due to education, retirement, or caring for young children, and the remainder (10/60) were manually employed—half on a skilled basis and half as unskilled workers. Ethnically, 90% of participants were white (54/60), 5% (3/60) were Asian, 3% (2/60) were Black or mixed race, and 2% (1/60) preferred not to say. Over three-quarters (47/60) claimed no connections with farming or the dairy industry, and 17% (10/60) said they did have connections, but not themselves or through close family. Regarding diet, the majority (85%, 51/60) said they consumed “most things” and 12% (7/60) were vegetarian; only one was vegan, and one was dairy-free. Most consumed cows' milk, but 10% (6/60) mainly or exclusively consumed plant-based alternatives.

While qualitative samples are not expected to represent the wider population in the same way as quantitative samples, it is worth noting variances against the demographics of the wider UK population. The sample comprised 34 women and 26 men (57 and 43% respectively) compared with a 52% women and 48% men gender balance within the wider population (68). Only 8% of the sample were ethnically non-White, making it less ethnically diverse than the UK population average, in which 14% identify as non-White. However, 63% of the sample which was found to be economically active (i.e., contributing to the national economy) was comparable with Government (Office of National Statistics) data, where 64% of the national population within the same age ranges was found to be economically active (69).

Within the interviews, almost all participants referred to having seen a cow in real life, and this was almost always outside in the environment (e.g., in surrounding fields, from car windows, during walks) or, less frequently, during visits to farms. Around half had first-hand memories and experiences of cows, farms, or farmers from childhood. Three mentioned either growing up or working on a farm, and a further eight claimed contact on more than one occasion with a specific farm or farmer, currently or in the past. A third of interviewees cited specific items they had seen in the news or media, many of which were about the low price of milk paid to farmers (although others raised financial hardships faced by farmers without offering a source). Three-quarters referenced a TV or radio program, book, film, or picture that had a connection with dairy farming. A dozen participants referred to animal rights or campaign group activities they were aware of, with seven making specific mention of campaign material they had watched or seen.

### Summary of frames

Through conducting frame analysis on interviewee data, we established that when describing their perceptions of dairy farming, most data within scope focused on the cow, as the key actor in dairy farming, and the farmer, as the keeper of the cow and therefore chief architect of her experiences. The cow was characterized through three diverse frames, and the farmer two, with the care of the cow at the hands of the farmer a key part of the latter. Several different but sometimes overlapping narratives within each frame added dimension and meaning.

Interviewees made sense of the cow through three frames characterized as Enduring Cow, Fellow Cow, and Force of Nature. All three frames served to indicate a connection between the participants and cows, albeit in different ways, which resulted in concern or interest in her well-being. Half of the participants comfortably held two frames at the same time, and a third, all three, suggesting the frames were facets of the same entity.

Participants viewed the farmer as a Traditional Farmer, and a Modernizing Farmer, with almost half of the participants expressing perceptions aligned with both frames. However, each of these frames was seen through positive and negative narratives. This duality stemmed from an almost universal acknowledgment among interviewees of the difficult position farmers find themselves in financially with some recognizing a potential knock-on impact on the care of the cow. Participants were generally sympathetic to the tough choices facing farmers—but how farmers responded to this financial pressure and how it impacted the cow were distinguishing factors between the positive and negative characterizations of each of these frames.

**Figure 1:**
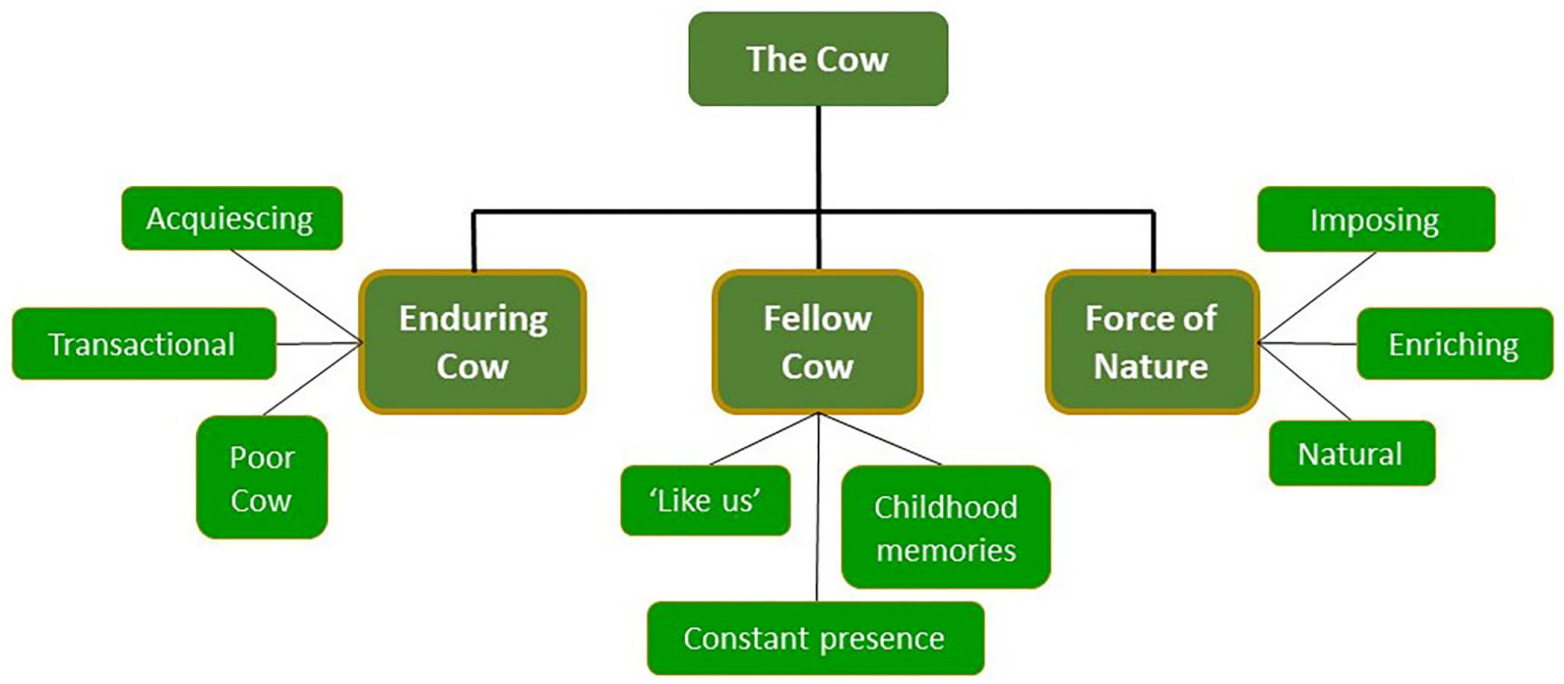
Summary of the different frames developed for the cow, and underlying “narratives” through which each frame was expressed.

**Figure 2:**
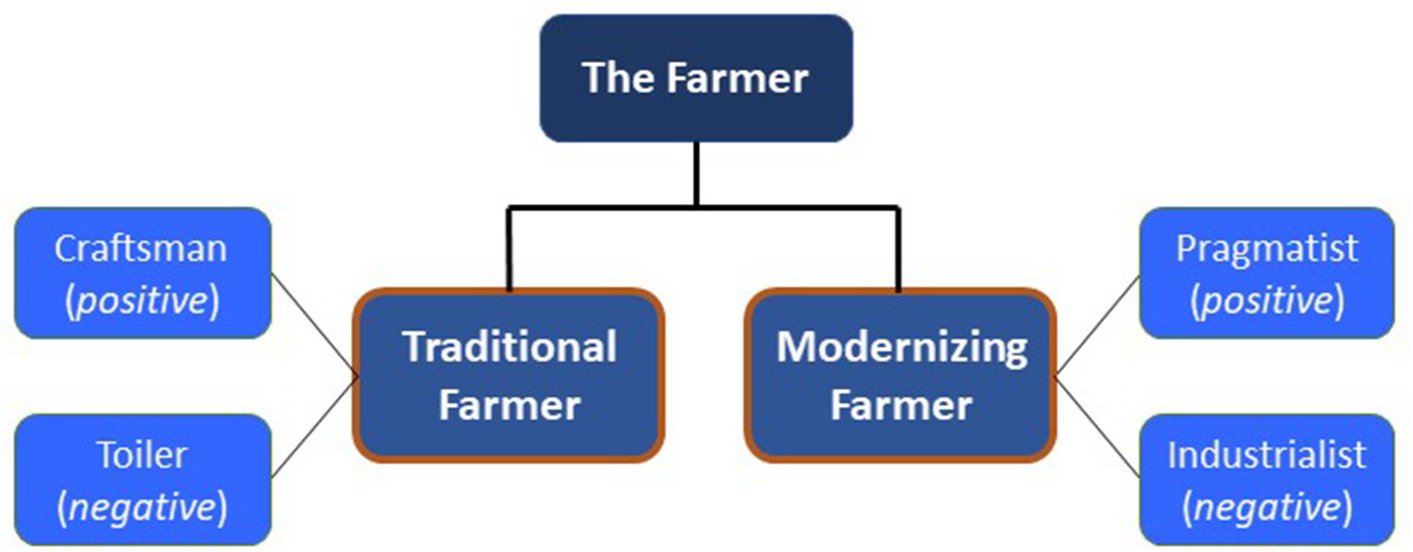
Summary of the different frames for the farmer and the underlying positive and negative “narratives” through which each frame was expressed.

Frames for the cow and their underlying narratives are summarized in Figure 1; frames for the farmer are similarly summarized in Figure 2. Anonymized coded excerpts from the interviews have been used to illustrate the findings.

### Framing “the cow”

#### Cow frame 1—Enduring Cow

The “Enduring Cow” frame, expressed by around two-thirds of interviewees, was characterized through underlying narratives describing: an acquiescing creature, committed to routine work; a participant in a transactional role deserving fair treatment; and an exploited and sometimes mistreated “poor cow” working in a job from which she cannot escape.

The acquiescent Enduring Cow was recognized for her suitability for work in the dairy herd and she was appreciated by some for her aptitude for applying herself. Many interviewees saw her as inured to her tasks, as observed here in a TV documentary:

*Participant 35: “…to see them coming in…they walk into the barn, and they know exactly what they're going to do, they're conditioned if you like to have their two hours milking. If you go to the farm when the farmer's due to pick up for milking, they're all congregating round the gate, they know what's coming.”*

The acquiescent Enduring Cow was characterized as having simple demands:

*Participant 22: “The lower end of Maslow's hierarchy of needs …They don't need much else. Don't anthropomorphize. They are not humans. They need welfare. I believe animals should have rights, but they don't vote, they don't think about the environment, they don't have these higher-level things.”*

However, some participants commented on her ability to adapt to technology such as milking parlors and new robotic milking systems.

*Participant 53: “Well you can almost train them, well you can, can't you, to know when they're gonna be milked and they walk toward the milking thing, and they stand there. It's all automized today, they know when they're gonna be—even when they're in the fields they know and they seem to start moving, don't they…”*

The transactional narrative within the Enduring Cow frame captured a sense of duty or moral responsibility felt by participants on behalf of the cow, recognizing that she was enduring work for their benefit and should be recognized for her “service”. The recompense included kind treatment and gentle handling.

*Participant 39: “I don't know how responsive they are to humans or how intelligent they are as an animal, but I imagine in my head if they're having a nice stroke and they're being talked to … it's a bit more personable … it's making the time or their life a little bit less as though they are on a production line.”*

There was also recognition that if the cow was able to trust the farmer or her handler, then that would make her experience bearable, if not rewarding.

*Participant 56: “I think having secure safe bonds is incredibly important, especially when those people are then doing stuff to you, so they're plugging you into things or they're making you go inside 'cause it's snowing or whatever, I think if you've got that trust and that bond then those experiences are gonna be very, very different to if you're afraid of someone or being forced to do something you don't know.”*

Through the narrative of “poor cow,” interviewees raised concerns about the Enduring Cow and unpleasant practices she might be subjected to that they had increasingly “heard of” or had seen—mainly through media and found perplexing in light of what they might have previously believed.

*Participant 31: “…there's a lot of things about local farms or it might be overseas …where they're talking about dairy cows and how they're poorly treated and how the calves are ripped away from the mothers and how the milk has got pus in it and it's disgusting.”*

*Participant 15: “…you can't see behind the scenes whether they're having their calves taken off them, where they're forced to get pregnant until they die just so they lactate…”*

While most references to distasteful dairy farm practices or poor welfare within this frame related to imagined, extrapolated, or curated imagery from third parties, the concerns of some interviewees had been corroborated by personal observation.

*Participant 41: “You see cows in a field, and you can see their rib bones but huge stomachs and they're literally struggling to walk because they've got so much milk.”*

Discomfort, stress, swelling, strain, exploitation, and exhaustion were some of the words used in this characterization of the Enduring Cow, and anxieties were expressed about routine dairy farming practices such as having to produce a calf every year and artificial insemination.

*Participant 50: “The cows were just tret* [treated] *awful, they're artificially inseminated pretty much all the time. The normal lifespan of a cow is 20-25 years, and they only live for five because they're constantly pregnant ….”*

#### Cow frame 2–Fellow Cow

Almost three-quarters of participants perceived the cow as a “Fellow Cow”—a colleague, peer, or equal with whom they were familiar and who had shared life journeys or experiences. The Fellow Cow was characterized through several different narratives: companionable childhood connections; a constant presence around them; and being “like us”—understanding the cow's life through their own. Many interviewees expressed feelings of a bond with the animal despite more than three-quarters (found through the questionnaire) having no connection with farming or the dairy industry.

Personal childhood memories played a significant role in building a feeling of fellowship. Cows in the environment around them from an early age were mentioned by many participants, especially seeing cows in fields during car or train journeys.

*Participant 45: “… we would've travelled past fields, and we always would've seen cows; we were always looking out for cows.”*

*Participant 36: “If you're ever driving by you always see black and white cows … just generally seeing them around, they were all round everywhere.”*

Some participants had farming relatives and fondly recalled visiting them and their cows as children. Emotional connections formed with cows appeared vivid in childhood accounts. Some were personal and others were the experiences of friends or relatives, which appeared just as relatable.

*Participant 57: “I was taught how to milk a cow. I must have been probably about five, six, seven years old but I will never forget the feeling… And then the cow is just standing there as if nothing happened. And she just lets you do anything as long as you don't pull too hard.”*

*Participant 37: “… my wife, she'll tell you…there's a lovely photograph of her and Jimmy Bullock…and she would go feed Jimmy…and talk to Jimmy Bullock—but Jimmy Bullock went to the slaughterhouse, you know, and that's the way it goes.”*

The Fellow Cow was also a constant and tangible presence beyond childhood, “always there” as if an anchor despite what else changed. Seeing cows “dotted” around fields, “littering” the countryside, or representing the passage of miles or time were common recollections:

*Participant 33: “…when I go back to Wales, I don't see the same cows obviously, I think they've long gone, but the same kind of picture is painted in my head as I'm driving back and the kids are in the back seat screaming out, they can see the cows and stuff.”*

By contrast, the absence of the cow from fields during serious disease outbreaks diminished the countryside and felt like a cultural loss:

*Participant 23: “I can remember when the big Foot and Mouth outbreaks were out and when they were burning cows in fields and how different our landscape looked without cows and sheep on the slopes … It wasn't nice and I didn't like it.”*

*Participant 5: “I remember during the Mad Cow Disease* [likely to be referring to Foot and Mouth Disease]*, going to the Lake District, and it was quite a peculiar feeling that all the fields were empty, because it enhances our countryside, it's our culture.”*

“Like us” was a third narrative through which the Fellow Cow was perceived—participants understood her world through comparisons with theirs. The cow's experiences of issues as diverse as digestive health, lactating, social life, and dealing with the weather were seen as if they were human experiences.

*Participant 14: “We've all got friends, we've all got colleagues; a farmer knows, he watches them every morning, some don't get on, Ermintrude don't get on with Gertrude.”*

*Participant 6: “I think if a cow is happy then it's going to give more milk, it's like a mother that if she's stressed there is a lot of reasons that she can't breastfeed her child. So I believe that if the cow is happy obviously it's going to produce more milk.”*

The cow's “working” life was also expressed as a parallel life to their own.

*Participant 11: “It always used to make me smile when I drove to work up the A45* [road] …*at certain times of the year you'd be driving along and the cows are heading for milking and they're literally walking in a line across field—there's no one there, they know it's milking time. And it used to make me smile, ‘Ah you're off to work as well'.”*

Cows were described as moving around to their agenda, determining where they go and what they do according to their dispositions, social lives, and whatever needs were being met. In this way, they appeared autonomous and similar to humans in terms of self-determination.

*Participant 1: “Because if I look at the cows that I see in the fields, the cows have got different personalities, you can see that, they're often quite spaced out…They move around between different fields of their own volition because the gates are open …that's why you can't tell which field you're going to see the cows in, they're often in one and then all of a sudden, they won't be there.”*

*Participant 55: “…from the way the cows have their own mind to go and milk* [through use of a robotic milker]*, that is natural...cows, from a manual milking point of view, they have to go to milk in the morning and the afternoon, whereas now they could be like at lunchtime, ‘Ooh, I quite fancy going to have a milk' and they can.”*

#### Cow frame 3—Force of Nature

More than half of the interviewees framed the cow as an elemental creature with a forceful, impactful presence, emphasizing her animal state and their inability to fully “access” or understand her. This “Force of Nature” frame was characterized by imposing scale and aggression, the way she enriched lives, sometimes through sensory stimulation, and how she appeared grounded in the natural world. The Force of Nature frame emphasized a distance from humans which some participants appeared to find intriguing.

Firstly, many participants referred to the scale of the cow—especially those to whom a lack of rural living experience made her size seem even more imposing and “real”.

*Participant 17: “I think the first time I saw a cow I was quite daunted by the size of it and great big udders*<*laughs*> *and it was all quite real you know? Compared to the plastic farm animals I had to play with but daunting in reality.”*

The “aggressive” side of her imposing nature implied unpredictability and was illustrated in several dangerous encounters.

*Participant 3: “I do remember being chased by a herd of cows on one occasion… And I'm not sure why they were chasing, we were able to get behind this fence and they just wandered off, but I do believe it's a very dangerous thing to be trampled.”*

This unpredictability was sometimes expressed as individuality and personality. Despite the implicit danger, stories were repeated as factual or amusing anecdotes rather than in fear, as if the experience was integral to being in the countryside or a badge of honor.

*Participant 1: “There was a story my mother told…she used to go in an evening to get the milkings from the farmer round the corner, so she would be perhaps six or seven at this stage. And there was a cow that they called ‘Dog' because it was a heifer and it guarded the farm gate and so she would go up, and she would have to go with a stick, because you show the stick, she said, ‘If you've got an aggressive cow just brandish a stick, a sizeable stick at it.' So, she always used to have to find a stick on the way there just in case this cow went for her.”*

The enriching sight, smell, sound, and touch of the cow, or the taste of her fresh milk, appeared to form particularly strong or visceral memories for participants within this elemental frame.

*Participant 7: “…we could watch them actually being milked and you could drink the milk and it always tasted different because it was fresh from the cow.”*

*Participant 57: “…the udder is so wonderfully soft. It's like velvet and so warm.”*

*Participant 10: “I have got memories of hearing those cows mooing …”*

*Participant 44: “They smell like butterscotch…”*

A further narrative in this frame was a characterization of the cow's embeddedness in nature and the rhythms of the natural world. Through her, watchers appeared to vicariously experience her peace or pleasure.

*Participant 40: “We go to Wales a lot. I've seen sheep out and cows and I've sometimes just stopped and looked at them and they seem to be liking it… They seem to be liking it, just a happy bunch of animals, you know?”*

*Participant 30: “I'd like to think in nice, big open fields sort of grazing away and mooing and in bliss, maybe on a night like this lots of mist coming out from everywhere.”*

Other interviewees referred to the way cows marked the natural turn of the seasons when herded to or from Alpine pasture or the symbolism of the cows being let out for the first time after the winter.

*Participant 46: “When they* [cows] *come out in the springtime, to us, it's like new life, like a new lamb, it's like new life to us, it signifies the start of the spring, it signifies to us that all is well.”*

*Participant 32: “…you let them out and they knew this, they'd jump and spring and they'd … it's as if they're happy to be let out.”*

### Framing “the farmer”

#### Farmer frame 1—the Traditional Farmer

Just over half of the participants described the frame of the “Traditional Farmer” as the farmer they had seen in children's books, in film, on TV, and sometimes on walks or car journeys. The farmer was imagined as older, male, born and bred a farmer, and often part of a farming family whose forebearers stretched back many generations. As a result, he had an ancestral commitment to the farm.

*Participant 18: “To put in a twee way, they're custodians of the land and as so many farms are handed down from father to son, they are custodians making sure that they hand it over in an even better condition than when they received it.”*

*Participant 19: “It would be the traditional aspects, it would be the cows in the field, then the cows in the barn, early morning milking, traditional elderly farmers who have had the farm for many generations and methods, tried, tested and almost sort of primitive.”*

The Traditional Farmer was seen to typically run a smaller farm, although when pressed, the definition of this provided by participants varied significantly, particularly in terms of the number of cows. It was implied by some that the Traditional Farmer only produced enough on his farm for his and his family's own immediate requirements rather than “mass farming”; selling products was not a prominent aspect of this frame. It was recognized that the smaller farm might not be financially viable, but the Traditional Farmer and his family might accommodate this by supplementing their income.

*Participant 18: “For many people the ideal is someone with a smallholding and five cows or something like that, but most people, you can't make a living like that, so for the people involved, they have to be able to make a living and you read about so many people now who are on a farm but their wife has to work off the farm, so that they can continue farming.”*

A strong sense of animal care was expressed and recognition of the Traditional Farmer's genuine bond or even love for his cows.

*Participant 6: “He's looking after the animals, if they're giving birth, he's got to help to give birth, he's got to look after them and make sure they're clean, feed them, if there's a problem, they will have to call the vets…I think that the farmer treats them as his children, and they've probably got names.”*

The farmer's skill with cows and commitment was often expressed in terms of him knowing his animals without having to refer to their ear tags or numbers, therefore knowing them as individuals.

*Participant 51: “He'd name all his cows, so that each cow had their own name. So I'd say it was more than a living to him, it was his lifestyle, but he genuinely cared about the animals as well.”*

*Participant 45: Yeah, it's a relationship, I think I would've pictured… just in my mind probably from films and things…they have a relationship with that animal whereas now they're just a number.”*

While personal handling and management of the cows was a recurring theme in this frame, with little reference to employed labor, it was understood the Traditional Farmer did use machinery, particularly milking machines, but this was often seen as traditional and as an enabler rather than detracting from the positive imagery.

*Participant 23: “There is the dairy farm that everybody looks at as idyllic and British with all the cows having individual names like Daisy and Buttercup and coming in for their milking twice a day in a nice, probably herringbone floored stall… you put the machine onto the teats and then the milk gets taken out of the cows.”*

In such a way, one underlying narrative for the Traditional Farmer was positive and nostalgic, as a “craftsman” or artisan. However, some recognized that their image of the Traditional Farmer might be idealized and were not oblivious to his shortcomings. For example, while the generational commitment to the family farm was admired, others thought this could trap family members who did not want to be there, or who did not care for the animals.

*Participant 47: “I know a lot of farms are inherited, they're passed through families, and I don't know many people that would choose to go into farming, it's such a difficult life, and as I said, in some cases for little reward.”*

*Participant 20: “… you might have the ancestors which were really passionate about looking after the animals, and it's just kind of been passed down the family and the younger people aren't so passionate about it.”*

Dirt, mud, dilapidation, and chaos were often connected with the Traditional Farmer but expressed as an integral and almost authentic if unfavorable aspect of the construct.

*Participant 10: “I think he was an oldish man is what I remember. …It was a family run farm, it felt like, with quite a dirty farmyard with cows wandering around.”*

*Participant 57: “So it's not very hygienic, obviously, all those flies and ugh, it was horrible. I never enjoyed a holiday there, but they are the experiences that I remember, and they are still fresh in my memory, like milking the cow.”*

The frame of the Traditional Farmer was also described as hard work physically and in terms of commitment and time invested.

*Participant 53: “I think it's hard work, dairy farming. They have to get up, those cows have got to be milked twice a day. They need looking after. I don't know how they make a profit…they've gotta look after them. I think it's hard work, 'cause round here it used to be a lot of farming, but now it's gone. I'm sure farms are dying out in this country; I'm quite convinced of it.”*

Underlying financial pressure was a commonly cited reason for this commitment, which was mostly attributed to poor prices for milk and the role of the supply chain.

*Participant 51: “Like I said, everybody has to earn a living, but I think the supermarkets have a lot on their shoulders... they want the price to be as low as possible and the person that suffers then is the farmer and then ultimately the animals.”*

Wider concern that poor financial viability would eventually impact the animals was expressed by several interviewees, albeit in a non-judgmental manner.

*Participant 23: “…the farmer might run out of money and hasn't got enough food to supplement them, or his silage has gone belly-up and he isn't able to feed them silage and he can't afford a vet… There are some very distressing things that appear in the papers—farmers that can't cope.”*

Thus, the negative narrative for the Traditional Farmer was more akin to a “toiler”—someone who needed to strive to maintain the farm in today's tough environment and, as a result, might not be able to fully cater to the cow's needs.

#### Farmer frame 2—the Modernizing Farmer

Almost three-quarters of interviewees framed dairy farmers as the “Modernizing Farmer”. In contrast to the Traditional Farmer, the Modernizing Farmer was adapting to tough market conditions and poor milk prices rather than having these externalities drive them under. An implicit part of this adaptation was a change in role from practical to managerial.

*Participant 39: “…in this day and age, I would imagine it's more about the commercial aspect and how they're gonna manage their buyers, rather than hands-on with the animals, just because I think probably everything's done by pumps and machines and things.”*

It was recognized that within the Modernizing Farmer frame, compromises sometimes had to be made between the care of the cow and surviving or making a profit, and this was broadly accepted by those who understood the challenging nature of the situation.

*Participant 12: “I think you've always got that welfare vs. profit balance and without knowing enormous amounts about it, it's difficult to know where the ideal balance would be…there's a trade-off, isn't there....”*

*Participant 54: “I feel like they're under pressure to make a wage and a living, so they've gotta decide what's best for them, what's gonna keep their farm afloat and what's gonna pay their bills might not necessarily benefit the cow.”*

How this balance was struck, the extent of the compromise between care of the cow and financial imperatives, and the reason behind it, appeared to be key factors as to whether this frame was perceived in a positive or negative light. Described positively, it was understood that the farmer's duty to the cow was maintained, as “doing right by her” was in the interests of both farmer and animal, even if that care was delivered in an unsentimental way. Within this generally constructive narrative, the Modernizing Farmer was accepted as a “pragmatist.”

*Participant 12: “I suppose it's in the farmer's interest to look after their animals' welfare, 'cause I guess that way the healthier they are the more they produce, and so I guess it's just the farmer's job to a large extent is kind of tending to that lifecycle…”*

*Participant 33: “So I don't get that warm fuzzy feeling that … he would go running into a field and be stroking the cattle and giving them pets…”*

It was broadly appropriate for “pragmatic” Modernizing farmers to make sufficient money for a decent living rather than for large profits; one interviewee suggested this might be because a responsible farmer would plow surplus profits back into the welfare of the animals.

*Participant 47: “I think a farmer, if they're making a good living from it, they're more likely to reinvest that and look after their—that's their livelihood—and look after their livestock. I think it's a good circle to set up.”*

However, if the cow's welfare was perceived to be traded off too readily or compromised for the sole reason of profit rather than survival or inability to cope, then the Modernizing Farmer assumed a more negative persona whose motivations became unsavory. In this way, the negative narrative surrounding the Modernizing Farmer was as an “industrialist,” who was utilizing the cow for their own benefit.

*Participant 15: “Trying to get the cows to produce as much as possible, not being particularly fussed if they get ill either, bunging loads of antibiotics in them or if they feel that they're not gonna be producing, they're too old or they're too sick then bunging them off to the knackers' yard as they call it.”*

*Participant 32: “…it could even be a businessman, not knowing anything about farming, and all he's wanting is to make as much money and that cow must produce ‘x' amount of milk, otherwise another cow must come in its place.”*

The Modernizing Farmer was generally viewed as having a larger farm relative to the Traditional Farmer, innovative or entrepreneurial, as having employed workers and being more mechanized. To help them cope with their growing managerial role or increased cow numbers, Modernizing Farmers were ready adopters of data tools and technology. The modern milking process was characterized by tubes, pumps, and wires; a number of interviewees envisaged long lines of cows and conveyor belts, or of them revolving on “rings” and platforms.

*Participant 50: “It's obviously that technology with them that's changed quite a bit as well 'cause it used to be just done by maids, doesn't it? But now I think they've got them udder clamps that milk the cows and things like that, and they're sterilized and everything like that, and there's a rotation of the cows coming into this thing to be milked for them to then go out and then another cow come in.”*

The advent of robotic milking or automated milking systems was a positive, “pragmatist” development in the eyes of most participants who mentioned them, feeling it was in step with what the cow would choose and therefore supported her autonomy.

*Participant 41: “So, the cows went in whenever they wanted to, there was like automatic teats and they were saying the automated process made it kinder for the cows because rather than being pulled in and then… manually getting the milk out, the cows would walk in when it felt natural to them.”*

Some of the adaptations “pragmatist” Modernizing Farmers made to cope with low milk prices were viewed as positive and innovative, in particular, diversification or adding value to products.

*Participant 37: “…I've seen the Countryfile* [TV program] *type thing where the farmer's been struggling, the young son has said, ‘OK dad, we've gotta do something about this otherwise we're gonna be out of business,' and they've gone over to making cheese… You have to diversify or die.”*

However, some of the Modernizing Farmer's modifications to the business were characterized negatively, as an “industrialist” approach, with increasing scale or numbers of cows viewed as a retrograde development, lessening the care of the cow.

*Participant 11: “…if you've got a thousand cows, you're not going to have the personal, semi-personal touch and you've got to rely on more people to do their job properly, you can't check all of them, you can't be in 30 fields at the same time and check that 50 cows over there and that 50 cows over there.”*

*Participant 31: “I think obviously once you get bigger and maybe your priorities are a bit different you either try and detach yourself or you just see it as a business opportunity, and you just don't have that emotional connection to your surroundings and to the animals that you're looking after.”*

Technology had downsides too, and these were expressed mainly as a loss of connection between the farmer and the cow.

*Participant 19: “In some respects it felt as though it was making the whole thing clinical and that there was no relationship between the cow and the farmer. The cow was more just an asset which was producing a product and it was … I suppose a bit like when I used to watch, or used to see the production lines of car assembly.”*

In a similar way, an increase in cow numbers was often associated with confining and stocking them more densely, and with the farm becoming more agribusiness, corporation, or company—in keeping with the negative “industrialist” narrative.

*Participant 42: “... it's gone for me like subsistence farming to profit, to capitalism basically. And as a result, you're seeing these massive industrial units ran by businesses looking for profit and you're ending up with these, like I said before, industrial scale operations with these big farms with big numbers of cattle…”*

Thus, the “pragmatist” narrative of the Modernizing Farmer might be described as “a farmer running a business,” vs. the “industrialist” narrative of “a businessman running a farm,” with almost two-thirds of participants evoking the former, just over half the latter.

#### Conflict between farmer frames

While many interviewees framed both cow and farmer in several different ways, within the farmer frames this caused friction, often giving rise to expressions of confusion or distrust which were not apparent within the cow frames. A manifestation of this was descriptions of the Traditional Farmer frame sometimes being dismissed as idealized or unrepresentative because they clashed with the Modernizing Farmer frame.

*Participant 14: “Well, I imagine from what I've seen and obviously Hugh Fearnley-Whittingstall* [celebrity chef]—*he's got his barns with his cows... I know that's not the real thing because obviously it's more intense than that, but that's what I imagine when you see a dairy farm. But obviously they are more enclosed.”*

This led to interviewees questioning whether the positive or negative manifestations were correct—specifically how cows were really kept in modern times, what they were fed or treated with, and who safeguarded them:

*Participant 55: “…big brands that collect the milk, they're responsible for how the farmer looks after the livelihood of the cow, would they take milk from a farmer that doesn't look after their cows? I don't know the answer to that.”*

Even participants who had previously felt confident about their positive perceptions of the care farmers took of their animals were finding it harder in recent times to be sure.

*Participant 31: “In some ways it makes me feel a bit sad because I feel that there's quite a growing element of people knocking all farming and doing that across the board without actually thinking, do you know what, it's a very broad spectrum and that a lot of farms are very strong on their welfare and their standards. But a part of me also thinks, well you know, which parts of these are true?”*

## Discussion

### Summary

In this study, we set out to develop an understanding of how dairy farming might be perceived, and the diversity of interpretive frames employed to form that perception. A better knowledge of these frames could provide farmers and veterinarians with improved insight into how the public characterizes dairy farming, why, and the impact this perception has. This in turn could help to establish more empathy and common ground between the dairy farming community and the public. Three original findings are suggested from the analysis:

1. The frames developed focus primarily on the cow and the farmer—but mainly on how the cow is cared for—indicating that the dairy cow and her care might lie at the center of perceptions about dairy farming.
2. Despite the general lack of experience or meaningful contact with dairy farming evident from the post-survey questionnaires, our participants relate to the dairy cow in a number of diverse ways; they feel a duty and moral responsibility for the Enduring Cow, a longstanding and instinctive familiarity with the heavily anthropomorphized Fellow Cow, and an elusive respect or longing for the experiences of the Force of Nature Cow.
3. The conflict between the different farmer frames and their underlying narratives may give rise to confusion or even distrust about the farmer's motives and their care of the cow, which is a key preoccupation among participants.

Together, these suggest the public judge the dairy farm (and therefore dairy farming) by the treatment of the cow; also, that they feel self-legitimized concern for the cow due to their perceived connections with her. To unpack these interpretations further, we will first consider how novel the frames identified within this study are against existing research, and then what the frames might signify for the dairy industry.

### Cow frames

The use and exploitation of the cow identified within the Enduring Cow frame is a commonly explored theme within the literature, echoing public concern about the impact of farm practices or conditions on animal welfare [e.g., (50, 53, 70). The way in which the cow is seen as a participant in an unspoken “contract” is also identified in Nijland et al. (38), and the concept that animal use in farming is acceptable provided the animal is fairly treated is consistent with the principles of the “human-animal contract” expressed by the Food Ethics Council (71), and more latterly in the ethical approach of “New Contractarianism” described in Hölker et al. (72).

The two other frames for the cow are less evident in the existing study of dairy farming and for this reason, their various aspects offer more unusual insights. These include the sensory perceptions of the cow within the Force of Nature frame, which were also suggested by visitors to dairy farms in Boogaard et al. (73). Also, within this frame, the enrichment our participants believe the cow provides to humans around her, conveying a sense of tranquility and peace, is reflected in Hassink et al.'s review of the therapeutic benefits of farm animals at care farms (74) and even in the recent emergence of “cow cuddling” where the public are offered opportunities to be comforted by embracing a cow (75, 76). Ideas that the cow is self-determining and generally “like us,” indicated within the Fellow Cow frame, have been examined in the literature previously, but mainly through the study of the human-animal bond between cow and farmer or worker, and the interaction of the cow with the machinery, processes, environment, and “work” of the farm. Examples of this include the cow's “collaboration” in the work of the farm (77), and the use of technology, ostensibly improving outcomes for man and animal yet causing increased alienation (78, 79). However, Kaarlenkaski's (80) study of the public's perceived relationships with cows, drawn from entries to a writing competition, offers useful insights from those mostly external to the industry; in this, cows were frequently portrayed by members of the public as active participants of human-animal interaction, and personal relationships with cattle were important to many of those submitting views (80).

In common with “imagining” rather than “knowing” the cow, anthropomorphism—where human traits are attributed to non-human entities—is evident across the Fellow Cow frame. Such perceived connections appear to have been extrapolated from and reinforced by the overt visibility of cows in fields, and the way in which people have entwined their lives with the cow, feeling familiarity through shared life experiences in childhood, the daily commute, holiday fun, etc. Anthropomorphism is not a new concept in philosophy or animal study, whether expressed by Aristotle in the 4th century BC (81), in the Romantic era of the late 18th and early 19th centuries (82) or in various published works throughout history (83). Despite this, anthropomorphism is commonly dismissed in livestock farming today—by farmers as sentimentalism (14) and by animal researchers as lacking scientific basis (84). Its use in the branding and marketing of dairy products can also be problematic in creating a falsified image of how foods are produced (85). However, anthropomorphism is also defended by others as an attempt by those with less knowledge to form connections with animals, and they suggest its judicious use offers opportunities for the public to build conceptual bridges with animals and think “with” them rather than just “about” them (86–88).

Finally, the contrast between the perceived familiarity of the Fellow Cow and the “otherness” of the Force of Nature has echoes in Ingold (89), Jones (90), and Berger (91). They observe that the evolution of modern farming has transformed our relationship with livestock. While we have increased the use of farm animals by enrolling them into our food systems and farm structures, this has changed them from autonomous and elusive beings into mere units of production. These authors suggest that deep down we still want our connections with animals to be on their terms, not ours, to experience their primitive connections with nature. Yet in our efforts to “know” them, we have turned them into artifacts. In our study, expressions of the cow's ubiquity and the anthropomorphic desire to bond with her, yet the reverence felt for her symbolic, cultural, and natural importance, indicates a similar tension—even if it is not consciously recognized by interviewees who hold these frames simultaneously.

All three frames indicate that the public perceives connections with the cow, whether through a moral responsibility for her well-being, a life traveled together, or a longing for her peace or naturalness; this has not been identified previously in literature to our knowledge.

### Farmer frames

Certain facets of the farmer frames we identified reflect previous studies. For example, the perception of kindness toward animals in the Traditional Farmer frame was found to be important to the public by Ellis et al. (92), Miele (93), and Weary and Robbins (94), and the view that animals on smaller farms have a better quality of life, better care and better chances to be managed as individuals is reflected in a range of studies (93, 95, 96). This association between attentive husbandry and the Traditional Farmer “type of farming” has also been leveraged in marketing through the use of fictitious farm names which suggest smaller operations that execute more “personalized” management of animals (97).

Equally, similar concerns around the Modernizing Farmer have been raised by Boogaard et al. (49) in their identification of unease within the public about the use of living beings for economic gain and progressive increases in farm size. The ambivalence with which the adoption of automation by the Modernizing Farmer was seen by our participants was typical of the positive (pragmatist) and negative (industrialist) narratives within the farmer frames. Concerns that technologies such as robotic milking could detach farmers from their cows have been identified previously (49, 98), but equally, the positivity about the potential animal welfare benefits that could arise echoed findings in Pfeiffer et al. (99). Participants who had seen actual robotic milking on farms or TV appeared largely supportive of the technology, reflecting conclusions in Millar et al. (100), which found more support for robotic milking technology among those with a better knowledge of the topic.

However, the most novel finding in the farmer frames was the way in which conflicting frames and their underlying narratives appeared to create confusion and distrust, leading many interviewees to doubt their formerly established views about farmers' motives and activities. For example, the Traditional Farmer, as described by a large number of interviewees, was stereotyped and nostalgic; many admitted this “craftsman” narrative was likely to be idealized—yet it was strongly held within this frame, possibly due to the lasting effect of childhood imagery from TV or books [e.g., (101), re. anchoring effect]. Conversely, while the Traditional Farmer was judged to have stronger bonds with the cow and thus deliver better care—echoing the bonds some participants themselves appeared to be seeking through the Fellow Cow frame—his lack of viability in the modern world as identified through the “toiler” narrative, was acknowledged as a welfare risk for the cow and thus reduced the trust placed in him.

Similarly, incompatible narratives surrounding the Modernizing Farmer frame caused uncertainty, even among those with more prior exposure to farming. On one hand, interviewees expressed positive personal experiences of innovative or expanding farmers using technology pragmatically to develop their farms without unduly compromising the welfare of the cow; these narratives jarred with powerful negative perceptions of the “industrialist” on the other hand, which appeared to reflect social media and documentary imagery they had seen, underlining commoditization, exploitation, and suffering of the cow. While first-hand experiences may have played an important role in framing, negative messages carry more salience (102) and therefore may have been hard for interviewees to ignore. The deciding factors for whether the Modernizing Farmer was seen as “good” or “bad” appeared to mainly rest on the motives of the farmer and the consequences on the cow. This resonates with Weary and Von Keyserlingk (28), who propose that the moral high ground many farmers adopt: “*I take care of the animals, the animals take care of me”* (103) is undermined in the eyes of the public when the narrative changes to: “*I provide care to the extent that this benefits me financially*.” Either way, these clashes undermine efforts by the dairy industry to explain its practices and generate trust and risk conveying fickleness.

These findings underline the importance of personal contact or experience with farmers, and of their overt demonstration of the “right” motives toward their animals. They also illustrate the way in which negative sources of information can fill gaps in memories and experiences to frame farmers in an altogether unsympathetic way.

### Other observations

It is noted that while some specific dairy farming practices were singled out by participants for comment, featuring especially within the Enduring Cow and “industrialist” Modernizing Farmer frames (for example, cow-calf separation, artificial insemination, and high milk yields), other prominent issues for the UK dairy industry (for example, bovine TB, and lameness) were barely raised, if at all. It could be that these merge into non-specific discomfort expressed with modern production systems. Alternatively, this could illustrate the disconnect between the dairy industry and the public over what constitutes welfare or good animal care (2, 104–106).

Broader issues such as the cultural loss of cows from fields during times of disease, low prices paid for milk, and wider social and economic changes that might, for example, mean children no longer want to take over the farm from their parents, were raised by a number of participants. Despite recognizing the negative impacts these might have on farm animal welfare, such events were acknowledged with a degree of sympathy and as largely outside farmers' control. Similar socioeconomic and sociocultural factors have been examined in relation to farmer engagement in the control of bovine TB (107), concluding that a raft of competing pressures explains farmers' withdrawal from the issue. That the public might understand the role such events play in a farmer's ability to deliver good cow care, particularly within the “toiling” Traditional Farmer frame, suggests they have a greater appreciation of the wider social, political, and economic framework farmers operate within than they are usually given credit for by farming communities (11–13).

### Industry learnings

Insight into the connections the wider population might have with the cow, and the confusion they feel over the motivations and actions of the farmer, offers the dairy industry an opportunity to alter its approach to address the current disconnect and build new bridges with the public. Such efforts have met with only limited success to date, and we hypothesize this is largely because the farming community often attempts to “educate” the public in the expectation this will satisfy concerns and lead to acceptance. However, addressing this perceived knowledge deficit, for example by taking the public to visit farms, often fails to change attitudes (108, 109). This is because it assumes, first, that the industry has full knowledge and understanding of public concerns when, in fact, they are likely to diverge (2), and second, that information will satisfy those concerns in the same way it would satisfy the concerns of a scientist or expert (110). Therefore, this model is flawed as it ignores diversity in knowledge, concerns, and objectives between those inside and those outside the industry.

Another challenge with this approach is the assumption that those outside the industry are the ones who must change—as indicated in the study from Benard and de Cock Buning (16), where public participants moved closer to the farmers' views about pig husbandry, but farmers did not reciprocate. Grunig and Grunig (111) describe this as asymmetric communication, where only the recipient of the information is expected to change, as opposed to symmetric communication which asks both parties to move position to reach a compromise.

However, the findings of this study suggest ways to build bridges—such as the dairy industry better acknowledging the connection the public feel toward the cow or doing more to exemplify her care and prioritization. Gaining a more robust social license through a co-ownership approach, as suggested by Broad (112), would recognize the vested interest the public has in the way the cow is managed; also termed “reflexive modernity,” this strategy moves us away from the idea that only farmers and veterinarians have the legitimacy to input into how the cow should be managed. The wider dairy industry may also have a role to play in brokering such a transformation, adjudicating information between the farming community and the public as well as driving change. Such a role is explained in *The Honest Broker; Making Sense of Science in Policy and Politics* (113), which examines different ways by which scientists can support decision-making. Although entities within the dairy industry are more “trusted expert” than scientists, retailers and processors already play an important role in raising standards and anticipating risk. This suggests an opportunity to go further in helping resolve these more value-laden arguments between the public and farming, increasing symmetry of communication and forging agreement on a more co-designed future for dairy farming.

### Study limitations

While efforts were made to ensure the interview sample was of an appropriate size and diversity to elicit a broad range of data to address the research question, the nature of the recruitment process and the method of data collection are likely to have favored those with more flexibility of time to attend an interview—although it is not known whether this would have materially impacted the results. The aim was to evenly represent all six citizen groups from Jackson et al. (47), but this was ultimately not the case, with representation ranging between seven participants from the least-represented group, to 15 from the most. Participants from rural populations were actively sought, but the definition of a rural population and a person with rural living experience either varies or is difficult to ascertain due to a lack of data. Ethnic minorities and the youngest age group were also under-represented within the sample. While the aim was not to create a sample that represented the broader population, it was to capture a breadth of data that—through frame analysis—would increase knowledge of the factors contributing to the “disconnect” between dairy farming and the public. Hence diversity was important. For example, more “generation Z” (born since 1995) participants might have produced valuable insight as to the framing of dairy farming that is resulting in changing dietary habits (114).

Lastly, our participants were members of the public from the UK; while there will be many similarities in attitude among people from countries with similar climates, economies, and dairy sectors, demographic and attitudinal differences are inevitable; results should therefore be extrapolated with caution.

## Conclusions

Through frame analysis, hitherto unappreciated connections the public feel for dairy cows have been identified, alongside confusion toward farmers and their care of the cow. These findings are novel and provide fresh insight to support the dairy farming community in taking an empathetic and informed approach to bridging the growing disconnect with the public.

## Data availability statement

The datasets presented in this article are not readily available due to the UK General Data Protection Regulation (UK GDPR). Requests to access the datasets should be directed to jasmeet.kaler@nottingham.ac.uk.

## Ethics statement

The studies involving human participants were reviewed and approved by University of Nottingham School of Veterinary Medicine and Science's Research Ethics Committee (No. 1860 160930). The patients/participants provided their written informed consent to participate in this study.

## Author contributions

JK led on study design and was responsible for the funding. AJ conducted the research, performed the analysis and drafted the manuscript with the guidance and oversight of JK and advisory support of MG. JK and MG reviewed the draft manuscript, advised of edits, and approved the final edit for submission. All authors contributed to the article and approved the submitted version.

## Conflict of interest

The authors declare that the research was conducted in the absence of any commercial or financial relationships that could be construed as a potential conflict of interest.

## Publisher's note

All claims expressed in this article are solely those of the authors and do not necessarily represent those of their affiliated organizations, or those of the publisher, the editors and the reviewers. Any product that may be evaluated in this article, or claim that may be made by its manufacturer, is not guaranteed or endorsed by the publisher.

## References

[1] Harrison R. Animal machines. London, UK: Vincent Stuart Ltd. (1964).

[2] de Greef KH Stafleu FR de Lauwere CC. A simple value-distinction approach aids transparency in farm animal welfare debate. J Agric Environ Ethics. (2006) 19:57–66. 10.1007/s10806-005-4527-1

[3] Eurobarometer. Attitudes of Europeans Towards Animal Welfare: Special Eurobarometer 442. Available online at: European Commission (2016). https://data.europa.eu/data/datasets/s2096_84_4_442_eng?locale=en (accessed September 25, 2020).

[4] Stannard S. Farmers continue to be the most trusted part of the supply chain, but there is continued demand for transparency. AHDB (2021). Available online at: https://ahdb.org.uk/news/consumer-insight-farmers-continue-to-be-the-most-trusted-part-of-the-supply-chain-but-there-is-continued-demand-for-transparency (accessed March 4, 2021).

[5] Rodak O. Hashtag hijacking and crowdsourcing transparency: social media affordances and the governance of farm animal protection. Agric Human Values. (2020) 37:281–94. 10.1007/s10460-019-09984-5

[6] Wonneberger A Hellsten IR Jacobs SHJ. Hashtag activism and the configuration of counterpublics: Dutch animal welfare debates on Twitter. Information, Commun Soc. (2020) 24:1–18. 10.1080/1369118X.2020.1720770

[7] Institute of Governmental Studies. Proposition 2. University of California, Berkeley. (2021).

[8] Ares E. Animal Sentience Brexit. (2019). Available online at: https://commonslibrary.parliament.uk/research-briefings/cbp-8155/.

[9] Darwent N Leaver C. UK's First Free Range Milk Launched Today. Free Range Dairy (2015). Available online at: http://www.freerangedairy.org/2015/07/uks-first-free-range-milk-launched-today/ (accessed March 10, 2019).

[10] White K. Morrisons to launch new high-welfare chicken range. The Grocer. (2021). Available online at: https://www.thegrocer.co.uk/sourcing/morrisons-to-launch-new-high-welfare-chicken-range/653641.article (accessed March 31, 2022).

[11] de Rooij SJG de Lauwere CC van der Ploeg JD. Entrapped in group solidarity? Animal welfare, the ethical positions of farmers and the difficult search for alternatives. J Environ Policy Plan. (2010) 12:341–61. 10.1080/1523908X.2010.528882

[12] Heleski CR Mertig AG Zanella AJ. Stakeholder attitudes toward farm animal welfare. Anthrozoos. (2006) 19:290–307. 10.2752/08927930678541543931837785

[13] Sumner CL von Keyserlingk MAG Weary DM. Perspectives of farmers and veterinarians concerning dairy cattle welfare. Anim Front. (2018) 8:8–13. 10.1093/af/vfx006 32002209 PMC6951867

[14] Stevens TM Aarts N Dewulf A. Using emotions to frame issues and identities in conflict: farmer movements on social media. Negot Conflict Manag Res. (2020) 0:1–19. 10.1111/ncmr.12177

[15] Albernaz-Gonçalves R Olmos G Hötzel MJ. My pigs are ok, why change?—animal welfare accounts of pig farmers. Animal. (2021) 15:154. 10.1016/j.animal.2020.100154 33573976

[16] Benard M de Cock Buning T. Exploring the potential of Dutch pig farmers and urban-citizens to learn through frame reflection. J Agric Environ Ethics. (2013) 26:1015–36. 10.1007/s10806-013-9438-y

[17] Smid A-MC de Jong S Inberg PHJ Sinclair S von Keyserlingk MAG Weary DM. Western Canadian dairy farmers' perspectives on the provision of outdoor access for dairy cows and on the perceptions of other stakeholders. J Dairy Sci. (2022). 10.3168/jds.2021-21237 35221071

[18] Ritter C Russell ER Weary DM von Keyserlingk MAG. Views of American animal and dairy science students on the future of dairy farms and public expectations for dairy cattle care: a focus group study. J Dairy Sci. (2021) 104:7984–95. 10.3168/jds.2020-19732 33896636

[19] Foster M. The Truth about Gestation Stalls. Hoosier Farm Babe Blog. (2012). Available online at: www.hoosierfarmbabe.com/2012/08/the-truth-about-gestation-stalls.html (accessed March 7, 2021).

[20] Morello V. Quebec Dairy Farmer her Cow Pals are an Instagram Hit. CBC (2019). Available online at: https://www.cbc.ca/news/canada/montreal/quebec-dairy-farmer-instagram-1.5208292 (accessed March 7, 2021).

[21] Hoggard A. Andrew Hoggard's address to the Dairy Council at Federated Farmers' National Conference. Federated Farmers of New Zealand. (2017). Available online at: www.fedfarm.org.nz (accessed March 7, 2021).

[22] Martin P Shepheard M. What is meant by the social licence? In:Williams J Martin P, editors. Defending the Social Licence of Farming: Issues, Challenges and New Directions for Agriculture. Collingwood, VIC, Australia: Csiro Publishing. (2012).

[23] Bashi Z McCullough R Ong L Ramirez M. Alternative Proteins: The Race for Market Share is on. McKinsey & Company (2019). Available online at: https://www.mckinsey.com/industries/agriculture/our-insights/alternative-proteins-the-race-for-market-share-is-on (accessed June 6, 2020)

[24] Stannard S. Consumer Focus: *The Rise of Plant-Based Food Products and Implications for Meat and Dairy*. AHDB (2018). Available online at: https://ahdb.org.uk/knowledge-library/consumer-insight-consumer-focus-the-rise-of-plant-based-food-products-and-implications-for-meat-and-dairy (accessed June 6, 2019).

[25] Kalte D. Political veganism: An empirical analysis of vegans' motives, aims, and political engagement. Polit Stud. (2021) 69:814–33. 10.1177/0032321720930179

[26] Schenk P Rössel J Scholz M. Motivations and constraints of meat avoidance. Sustain. (2018) 10:1–19. 10.3390/su10113858

[27] Ruder Finn. Activism Goes Mainstream: A Look at who's Taking Action and Why. London, UK (2019).

[28] Weary DM von Keyserlingk MAG. Public concerns about dairy-cow welfare: How should the industry respond? Anim Prod Sci. (2017) 57:1201–9. 10.1071/AN16680

[29] Shmueli D Elliott M Kaufman S. Frame changes and the management of intractable conflict. Confl Resolut Q. (2006) 24:207–18. 10.1002/crq.169

[30] Goffman E. Frame Analysis: An Essay on the Organization of Experience. New York: Harper & Row. (1974).

[31] Bartlett F. Remembering: A Study in Experimental and Social Psychology. London, UK: Cambridge University Press. (1932).

[32] Minsky M. A Framework for representing knowledge. In:Winston P, editor. The Psychology of Computer Vision. New York: McGraw-Hill (1975). p. 211–77.

[33] Aukes EJ Bontje LE Slinger JH. Narrative and frame analysis: Disentangling and refining two close relatives by means of a large infrastructural technology case. Forum Qual Sozialforschung. (2020) 21:Art. 28. 10.17169/fqs-21.2.3422

[34] Aarts MN Van Woerkum CM. F*rame Construction in Interaction*. In: Engagement. 12th MOPAN International Conference. Pontypridd, UK. (2006). p. 229–37.

[35] Dewulf A Gray B Putnam L Lewicki R Aarts N Bouwen R. Disentangling approaches to framing in conflict and negotiation research: a meta-paradigmatic perspective. Hum Relat. (2009) 62:155–93. 10.1177/0018726708100356

[36] Virkki T Husso M Notko M Holma J Laitila A Mäntysaari M. Possibilities for intervention in domestic violence: frame analysis of health care professionals' attitudes. J Soc Serv Res. (2015) 41:6–24. 10.1080/01488376.2014.917449

[37] van Lieshout M Aarts N. Outside is where it's at!: youth and immigrants' perspectives on public spaces. Sp Cult. (2008) 11:497–513. 10.1177/1206331208320493

[38] Nijland HJ Aarts N van Woerkum CMJ. Exploring the framing of animal farming and meat consumption: On the diversity of topics used and qualitative patterns in selected demographic contexts. Animals. (2018) 8:ani8020017. 10.3390/ani8020017 29364860 PMC5836025

[39] Vigors B. Citizens' and farmers' framing of “positive animal welfare” and the implications for framing positive welfare in communication. Animals. (2019) 9:1–22. 10.3390/ani9040147 30987330 PMC6523948

[40] Shortall O Ruston A Green M Brennan M Wapenaar W Kaler J. Broken biosecurity? Veterinarians' framing of biosecurity on dairy farms in England. Prev Vet Med. (2016) 132:20–31. 10.1016/j.prevetmed.2016.06.001 27664445

[41] Braun V Clarke V. Successful Qualitative Research: A Practical Guide for Beginners. London, UK: SAGE Publications Ltd. (2013).

[42] Tavakol M Sandars J. Quantitative and qualitative methods in medical education research: AMEE guide No 90: part II. Med Teach. (2014) 36:838–48. 10.3109/0142159X.2014.915297 24845954

[43] Gill P Stewart K Treasure E Chadwick B. Methods of data collection in qualitative research: interviews and focus groups. Br Dent J. (2008) 204:291–5. 10.1038/bdj.2008.192 18356873

[44] Ng SL Baker L Cristancho S Kennedy TJ Lingard L. Qualitative research in medical education: Methodologies and methods. In:Swanwick T Forrest K O'Brien BC, editors. Understanding Medical Education: Evidence, Theory, and Practice. 3rd ed. John Wiley & Sons, Incorporated (2018). p. 427–41.

[45] While A Barriball KL. Collecting data using a semi-structured interview: a discussion paper. J Adv Nurs. (1994) 19:328–35. 10.1111/j.1365-2648.1994.tb01088.x 8188965

[46] Etikan I Musa SA Alkassim RS. Comparison of convenience sampling and purposive sampling. Am J Theor Appl Stat. (2016) 5:1–4. 10.11648/j.ajtas.20160501.1124899564

[47] Jackson A Green M Millar K Kaler J. Is it just about grazing? UK citizens have diverse preferences for how dairy cows should be managed. J Dairy Sci. (2020) 103:3250–63. 10.3168/jds.2019-17111 32057434

[48] Boogaard BK Oosting SJ Bock BB. Elements of societal perception of farm animal welfare: a quantitative study in The Netherlands. Livest Sci. (2006) 104:13–22. 10.1016/j.livsci.2006.02.010

[49] Boogaard BK Bock BB Oosting SJ Wiskerke JSC van der Zijpp AJ. Social acceptance of dairy farming: the ambivalence between the two faces of modernity. J Agric Environ Ethics. (2011) 24:259–82. 10.1007/s10806-010-9256-4

[50] Kendall HA Lobao LM Sharp JS. Public concerns with animal-well-being: place, social structural location, and individual experience. Rural Sociol. (2006) 71:399–428. 10.1526/003601106778070617

[51] Vanhonacker F Verbeke W van Poucke E Tuyttens FAM. Segmentation based on consumers' perceived importance and attitude toward farm animal welfare. Int J Sociol Food Agric. (2007) 15:84–100.

[52] Vanhonacker F van Poucke E Tuyttens F Verbeke W. Citizens' views on farm animal welfare and related information provision: exploratory insights from Flanders, Belgium. J Agric Environ Ethics. (2010) 23:551–69. 10.1007/s10806-010-9235-9

[53] Cornish A Raubenheimer D McGreevy P. What we know about the public's level of concern for farm animal welfare in food production in developed countries. Animals. (2016) 6:74. 10.3390/ani6110074 27854336 PMC5126776

[54] Rentfrow PJ Jokela M Lamb ME. Regional personality differences in Great Britain. PLoS ONE. (2015) 10:1–20. 10.1371/journal.pone.0122245 25803819 PMC4372610

[55] Malterud K Siersma VD Guassora AD. Sample size in qualitative interview studies: guided by information power. Qual Health Res. (2016) 26:1753–60. 10.1177/1049732315617444 26613970

[56] Braun V Clarke V. Thematic analysis: a practical guide. London, UK: SAGE Publications Ltd. (2022).

[57] Britten N. Qualitative interviews in medical research. Br Med J. (1995) 311:251–3. 10.1136/bmj.311.6999.251 7627048 PMC2550292

[58] Ritchie J Lewis J Elam G. Designing and selecting samples. In:Ritchie J Lewis J, editors. In; *Qualitative Research Practice: A Guide for Social Science Students and Researchers*. London, UK: Sage (2003). p. 77–108.

[59] Vasileiou K Barnett J Thorpe S Young T. Characterising and justifying sample size sufficiency in interview-based studies: systematic analysis of qualitative health research over a 15-year period. BMC Med Res Methodol. (2018) 18:1–18. 10.1186/s12874-018-0594-7 30463515 PMC6249736

[60] McMullin C. Transcription and qualitative methods: implications for third sector research. Voluntas. (2021). 10.1007/s11266-021-00400-3. [Epub ahead of print]. 34522070 PMC8432276

[61] Bourke B. Positionality: reflecting on the research process. Qual Rep. (2014) 19:1–9. 10.46743/2160-3715/2014.102631139407

[62] Darwin Holmes AG. Researcher positionality—a consideration of its influence and place in qualitative research—a new researcher guide. Shanlax Int J Educ. (2020) 8:1–10. 10.34293/education.v8i4.3232

[63] Nederhof AJ. Methods of coping with social desirability bias: a review. European J Soc Psychol. (1985) 15:263–80. 10.1002/ejsp.2420150303

[64] Larson RB. Controlling social desirability bias. Int J Mark Res. (2019) 61:534–47. 10.1177/1470785318805305

[65] Björnehed E Erikson J. Making the most of the frame: developing the analytical potential of frame analysis. Policy Stud. (2018) 39:109–26. 10.1080/01442872.2018.1434874

[66] Freeman M. Constant comparative method. In:Mathison S, editor. Encyclopedia of Evaluation. Thousand Oaks, CA: SAGE Publications Inc. (2011).

[67] Saldaña JM. The Coding Manual for Qualitative Researchers. Third London, UK: SAGE Publications Inc. (2015).

[68] Office of National Statistics. Overview of the UK population 2017. Available online at: https://www.ons.gov.uk/peoplepopulationandcommunity/populationandmigration/populationestimates/articles/overviewoftheukpopulation/july2017 (accessed February 22, 2019).

[69] Office of National Statistics. Employment, Unemployment and Economic Inactivity for People Aged 16 and Over and Aged From 16 To 64 2022. Available online at: http://www.ons.gov.uk/employmentandlabourmarket/peopleinwork/employmentandemployeetypes/datasets/employmentunemploymentandeconomicinactivityforpeopleaged16andoverandagedfrom16to64seasonallyadjusteda02sa (accessed March 2, 2022).

[70] Vanhonacker F Verbeke W van Poucke E Pieniak Z Nijs G Tuyttens F. The concept of farm animal welfare: citizen perceptions and stakeholder opinion in Flanders, Belgium. J Agric Environ Ethics. (2012) 25:79–101. 10.1007/s10806-010-9299-6

[71] Food Ethics Council. Farming Animals for Food: Towards a Moral Menu. London, UK. (2001). Available online at: https://www.foodethicscouncil.org/Resource/Farming-Animals-for-Food-towards-a-Moral-Menu/ (accessed June 24, 2021).

[72] Hölker S von Meyer-Höfer M Spiller A. Animal ethics and eating animals: consumer segmentation based on domain-specific values. Sustainability. (2019) 11:1–17. 10.3390/su11143907

[73] Boogaard BK Bock BB Oosting SJ Krogh E. Visiting a farm: an exploratory study of the social construction of animal farming in Norway and the Netherlands based on sensory perception. Int J Sociol Agric Food. (2010) 17:24–50.

[74] Hassink J de Bruin SR Berget B Elings M. Exploring the role of farm animals in providing care at care farms. Animals. (2017) 7:1–20. 10.3390/ani7060045 28574435 PMC5483608

[75] Pullman L. America's Latest Stress-Buster: Cuddling a Cow. Sunday Times. (2021). Available online at: https://www.thetimes.co.uk/article/americas-latest-stress-buster-cuddling-a-cow-gdn30rrq9 (accessed March 15, 2021).

[76] Gormly KB. Cow Cuddling has Become a Thing for Lonely Hearts in the Pandemic. Washington Post (2021). Available online at: https://www.washingtonpost.com/lifestyle/2021/03/08/cow-cuddle-sanctuary-covid/ (accessed June 6, 2021).

[77] Porcher J Schmitt T. Dairy cows: workers in the shadows? Soc Anim. (2012) 20:39–60. 10.1163/156853012X614350

[78] Holloway L. Subjecting cows to robots: Farming technologies and the making of animal subjects. Environ Plan D Soc Sp. (2007) 25:1041–60. 10.1068/d77j

[79] Hansen P. Becoming bovine: Mechanics and metamorphosis in Hokkaido's animal-human-machine. J Rural Stud. (2014) 33:119–30. 10.1016/j.jrurstud.2013.02.001 32288170 PMC7127193

[80] Kaarlenkaski T. Communicating with the cow: Human–animal interaction in written narratives. Nature, Cult Lit. (2014) 10:189–216. 10.1163/9789401210720_009

[81] Aristotle. The history of animals. In:Kalof L Fitzgerald A, editors. The Animals Reader: The Essential Classic and Contemporary Writings. 1st ed., Oxford, UK: Berg Publishers (2007). p. 5–7.

[82] Oerlemans OD. “The Meanest Thing that Feels”: Anthropomorphizing Animals in Romanticism. Mosaic. (1994) 27:1–32.

[83] Franklin A. “Good to think with”: theories of human-animal relations in modernity. In: Animals and Modern Cultures: A Sociology of Human-Animal Relations in Modernity. 1st, ed., London, UK: Sage (1999). p. 9–33. 10.4135/9781446217764.n2

[84] Wynne CDL. The perils of anthropomorphism. Nature. (2004) 428:606. 10.1038/428606a 15071579

[85] Stevens L Kearney M Maclaran P. Uddering the other: androcentrism, ecofeminism, and the dark side of anthropomorphic marketing. J Mark Manag. (2013) 29:158–74. 10.1080/0267257X.2013.764348

[86] Daston L Mitman G. Thinking with animals: New perspectives on anthropomorphism. J Hist Biol. (2005) 38:624–6.

[87] Buller H Morris C. Farm animal welfare: a new repertoire of nature-society relations or modernism re-embedded? Sociol Ruralis. (2003) 43:216–37. 10.1111/1467-9523.00242

[88] Philo C Wilbert C. Animal Spaces-beastly-places: new geographies of human-animal relations. London, UK: Routledge. (2000).

[89] Ingold T. Introduction. In:Ingold T, editor. What is an Animal? 1st ed., London, UK: Routledge (1988). p. 1-22.

[90] Jones O. “The restraint of beasts”: rurality, animality, actor network theory and dwelling. In:Cloke P, editor. Country Visions. Harlow, UK: Pearson Education Limited (2003). p. 283–307.

[91] Berger J. Why Look at Animals? In:Kalof L Fitzgerald A, editors. The Animals Reader: The Essential Classic and Contemporary Writings. 1st ed., Oxford, UK: Berg Publishers (2007). p. 251–61.

[92] Ellis KA Billington K McNeil B McKeegan DEF. Public opinion on UK milk marketing and dairy cow welfare. Anim Welf. (2009) 18:267–82.

[93] Miele M. Report Concerning Consumer Perceptions and Attitudes Towards Farm Animal Welfare. European Animal Welfare Platform, Brussels, Belgium. (2010).

[94] Weary DM Robbins JA. Understanding the multiple conceptions of animal welfare. Anim Welf. (2019) 28:33–40. 10.7120/09627286.28.1.033

[95] Lassen J Sandøe P Forkman B. Happy pigs are dirty! Conflicting perspectives on animal welfare. Livest Sci. (2006) 103:221–30. 10.1016/j.livsci.2006.05.008

[96] Lusk JL Norwood FB Prickett RW. Consumer preferences for farm animal welfare: Results of a nationwide telephone survey. Department of Agricultural Economics Oklahoma State University. (2007).

[97] The Week. Supermarket “Fake Farms” to Look Out for. (2017). Available online at: https://www.theweek.co.uk/87683/supermarket-fake-farms-to-look-out-for (accessed June 28, 2021).

[98] Schillings J Bennett R Rose DC. Exploring the potential of precision livestock farming technologies to help address farm animal welfare. Front Anim Sci. (2021) 2:1–17. 10.3389/fanim.2021.639678

[99] Pfeiffer J Gabriel A Gandorfer M. Understanding the public attitudinal acceptance of digital farming technologies: a nationwide survey in Germany. Agric Human Values. (2021) 38:107–28. 10.1007/s10460-020-10145-2

[100] Millar KM Tomkins SM White RP Mepham TB. Consumer attitudes to the use of two dairy technologies. Br Food J. (2002) 104:31–44. 10.1108/00070700210418721

[101] Tversky A Kahneman D. Judgment under uncertainty: heuristics and biases. Science. (1974) 185:1124–31. 10.1126/science.185.4157.1124 17835457

[102] Richey MH Koenigs RJ Richey HW Fortin R. Negative salience in impressions of character: Effects of unequal proportions of positive and negative information. J Soc Psychol. (1975) 97:233–41. 10.1080/00224545.1975.9923343 1207090

[103] Rollin BE. Animal production and the new social ethic for animals. J Soc Philos. (1994) 25:71–83. 10.1111/j.1467-9833.1994.tb00349.x

[104] von Keyserlingk MAG Weary DM. Stakeholder views, including the public, on expectations for dairy cattle welfare. WCDS Adv Dairy Technol. (2016) 28:147–58.

[105] Weary DM Schuppli CA Ventura B von Keyserlingk MAG. attitudes to contentious practices in dairy farming. WCDS Adv Dairy Technol. (2012) 24:371–82.

[106] Neave HW Sumner CL Henwood RJT Zobel G Saunders K Thoday H. Dairy farmers' perspectives on providing cow-calf contact in the pasture-based systems of New Zealand. J Dairy Sci. (2022) 105:453–67. 10.3168/jds.2021-21047 34696913

[107] Robinson PA. Farmers and bovine tuberculosis: contextualising statutory disease control within everyday farming lives. J Rural Stud. (2017) 55:168–80. 10.1016/j.jrurstud.2017.08.009

[108] Boogaard BK Oosting SJ Bock BB. Defining sustainability as a socio-cultural concept: Citizen panels visiting dairy farms in the Netherlands. Livest Sci. (2008) 117:24–33. 10.1016/j.livsci.2007.11.004

[109] Ventura BA von Keyserlingk MAG Wittman H Weary DM. What difference does a visit make? Changes in animal welfare perceptions after interested citizens tour a dairy farm. PLoS ONE. (2016) 11:1–18. 10.1371/journal.pone.0154733 27243965 PMC4887196

[110] Brunk CG. Public knowledge, public trust: Understanding the “knowledge deficit”. Community Genet. (2006) 9:178–83. 10.1159/000092654 16741347

[111] Grunig JE & Grunig LA. Models of public relations and communication. In:Grunig JE, editor. Excellence in Public Relations and Communication Management. Mahwah, New Jersey: Lawrence Erlbaum Associates Inc. (1992) p. 285–325.

[112] Broad GM. Animal Production, Ag-gag Laws, and the social production of ignorance: exploring the role of storytelling. Environ Commun (Internet). (2016) 10:43–61. 10.1080/17524032.2014.968178

[113] Pielke J. Roger A. The Honest Broker: Making Sense of Science in Policy and Politics. Cambridge, UK: Cambridge University Press. (2007). 10.1017/CBO9780511818110

[114] Food Standards Agency. The Future Consumer: Food Generation Z. London, UK: Food Standards Agency (2020). Available online at: https://www.food.gov.uk/sites/default/files/media/document/generation-z-full-report-final.pdf (accessed February 22, 2022).

