# Supplementary material for: Fellow cows and conflicting farmers: Public perceptions of dairy farming uncovered through frame analysis
Source: Front Vet Sci. 2022 Nov 17;9:995240. doi: 10.3389/fvets.2022.995240 (PMC9714478; doi:10.3389/fvets.2022.995240)
Supplement: Supplementary file 1 [file Table_1.docx]

**Key characteristics of participants in the study, by ascending age**

| **Age** | **Gender** | **UK Country/ English Region of Residence** | **Area Mainly Lived** | **Area Now Living In** | **Last visited a farm** |
| --- | --- | --- | --- | --- | --- |
| 19 | Female | North West England | Suburban | Suburban | Never |
| 22 | Male | North East & Yorkshire | Urban | Urban | >5 years ago |
| 22 | Male | North West England | Rural/country village | Urban | 1-5 years ago |
| 22 | Female | SE England/London | Mix of areas inc. rural | Rural/country village | Never |
| 23 | Male | North East & Yorkshire | Urban | Urban | Never |
| 24 | Male | West Midlands | Suburban | Suburban | 1-5 years ago |
| 25 | Female | North West England | Rural/country village | Urban | 1-5 years ago |
| 27 | Female | East Midlands | Mix of areas inc. rural | Rural/country village | Within the last year |
| 28 | Female | East Midlands | Suburban | Suburban | >5 years ago |
| 29 | Male | North East & Yorkshire | Suburban | Suburban | Within the last year |
| 30 | Female | Scotland | Suburban | Suburban | >5 years ago |
| 31 | Female | East Anglia | Rural/country village | Rural/country village | 1-5 years ago |
| 31 | Female | North East & Yorkshire | Rural/country village | Rural/country village | Within the last year |
| 31 | Female | Northern Ireland | Suburban | Suburban | 1-5 years ago |
| 32 | Male | North East & Yorkshire | Urban | Urban | >5 years ago |
| 32 | Female | Wales | Urban | Urban | >5 years ago |
| 33 | Female | East Anglia | Mix of areas not rural | Suburban | Within the last year |
| 33 | Female | North West England | Suburban | Suburban | Never |
| 34 | Female | SE England/London | Suburban | Suburban | >5 years ago |
| 34 | Female | West Midlands | Suburban | Suburban | >5 years ago |
| 35 | Male | SE England/London | Suburban | Suburban | >5 years ago |
| 36 | Male | North East & Yorkshire | Suburban | Suburban | >5 years ago |
| 36 | Female | SE England/London | Rural/country village | Rural/country village | Never |
| 37 | Female | SE England/London | Rural/country village | Rural/country village | Within the last year |
| 39 | Male | SE England/London | Urban | Urban | Never |
| 40 | Female | West Midlands | Suburban | Suburban | >5 years ago |
| 41 | Male | North East & Yorkshire | Urban | Suburban | >5 years ago |
| 41 | Female | SE England/London | Mix of areas inc. rural | Rural/country village | Within the last year |
| 43 | Male | East Anglia | Mix of areas inc. rural | Suburban | 1-5 years ago |
| 44 | Female | SE England/London | Suburban | Urban | >5 years ago |
| 45 | Female | SE England/London | Mix of areas inc. rural | Rural/country village | Within the last year |
| 48 | Male | East Midlands | Rural/country village | Rural/country village | >5 years ago |
| 48 | Female | North West England | Mix of areas inc. rural | Rural/country village | 1-5 years ago |
| 48 | Male | SE England/London | Urban | Urban | 1-5 years ago |
| 49 | Male | Northern Ireland | Mix of areas inc. rural | Rural/country village | >5 years ago |
| 49 | Male | Scotland | Urban | Urban | >5 years ago |
| 49 | Male | West Midlands | Mix of areas inc. rural | Urban | Never |
| 51 | Male | SE England/London | Suburban | Suburban | >5 years ago |
| 52 | Male | North East & Yorkshire | Suburban | Suburban | >5 years ago |
| 52 | Male | SE England/London | Rural/country village | Urban | Within the last year |
| 52 | Female | West Midlands | Urban | Urban | >5 years ago |
| 53 | Female | SE England/London | Suburban | Suburban | >5 years ago |
| 53 | Male | SE England/London | Mix of areas inc. rural | Urban | >5 years ago |
| 53 | Female | West Midlands | Mix of areas inc. rural | Suburban | Never |
| 57 | Female | East Anglia | Urban | Rural/country village | 1-5 years ago |
| 58 | Male | North East & Yorkshire | Mix of areas inc. rural | Rural/country village | Within the last year |
| 59 | Female | Scotland | Suburban | Suburban | >5 years ago |
| 59 | Female | SE England/London | Suburban | Suburban | >5 years ago |
| 60 | Male | Northern Ireland | Rural/country village | Rural/country village | Within the last year |
| 62 | Female | North West England | Suburban | Urban | 1-5 years ago |
| 62 | Female | Wales | Urban | Rural/country village | Within the last year |
| 63 | Female | SE England/London | Suburban | Suburban | >5 years ago |
| 66 | Male | SE England/London | Suburban | Suburban | >5 years ago |
| 66 | Male | SE England/London | Urban | Urban | Within the last year |
| 68 | Male | Wales | Urban | Rural/country village | Never |
| 71 | Female | Scotland | Urban | Urban | Never |
| 71 | Female | SE England/London | Suburban | Suburban | Never |
| 71 | Female | SE England/London | Suburban | Suburban | >5 years ago |
| 72 | Female | Wales | Mix of areas inc. rural | Suburban | Within the last year |
| 75 | Male | East Midlands | Mix of areas inc. rural | Rural/country village | >5 years ago |
