# Supplementary material for: Fellow cows and conflicting farmers: Public perceptions of dairy farming uncovered through frame analysis
Source: Front Vet Sci. 2022 Nov 17;9:995240. doi: 10.3389/fvets.2022.995240 (PMC9714478; doi:10.3389/fvets.2022.995240)
Supplement: Supplementary file 2 [file Data_Sheet_1.docx]

**Interview script**

**First of all, can we talk about your last food shopping trip – please can you describe the experience?**

Where did you go? Take me through it…

What did you look at? What did you buy?

Did you visit any of the dairy aisles? Would you have normally? What for?

**Now I want to turn to dairy farming. If I ask you to think of a dairy farm, what is the first image that comes to mind?**

How big is the farm?

Where do the cows live? What do they eat and where do they sleep?

What is the farmer doing?

Where does your image come from (– something you experienced yourself, or saw on TV, or read about)?

**I’m now going to show you three dairy farms you might find in the UK. I’m interested in your views on all three – do you have any questions about them? What thoughts come to mind?**

Describe how they match or differ from your image?

Was anything familiar, and if so, why?

Describe anything that appealed to you, or surprised you, or made you feel uncomfortable – and why.

***Inside / outdoors***

Describe what you think a cow experiences when she’s inside

What about experiences when she’s outdoors?

***Grazing***

If I said ‘graze’, what would you understand by it?

Imagine a cow grazing – what do you picture? What is the cow doing? How is she acting?

***Welfare***

What does ‘welfare’ mean to you?

How would you describe a cow with good or bad welfare?

How would you score each scenario out of 10 for cow welfare? (1 lowest, 10 highest). Why? What would make it a 10?

***Comfort***

And what do you imagine by ‘cow comfort’?

How would you rate the three scenarios for cow comfort, out of 10 with 1 having least comfort and 10 having most? Why? What would make it a 10?

***Summary***

Do you think grazing links in any way to welfare, or to the comfort of the cow? Why?

How might the comfort or welfare of the cow impact the farmer or the milk?

**Now I want you to think about these scenarios from a farmer’s perspective. What comes to mind?**

Why might you choose these different ways to keep your cows?

If you were running these farms, how would you enhance welfare in each one?

As a farmer how would you improve ‘cow comfort’ in each?

Is the environment a consideration? In what ways?

**If you were buying milk from these farms, would you have a preferred option? And would you pay more for it? How much?**

**Thanks for your views on that – We’re finished with these now so I’m going to put them aside.**

**Now I want to move on to talk about ‘naturalness’. How would you would describe ‘natural’?**

***General and food***

What words describe ‘natural’?

Can you describe the opposite of natural? (Would you call that unnatural or non-natural?)

In context of food, can you tell me about something natural?

Is ‘natural’ always good? Why? When is it not?

Can you describe any situations when non/unnatural is good?

***Farms & farm animals***

How would you apply ‘natural’ and ‘non/unnatural’ terms to farm animals?

In what ways is the act of people farming animals natural or non/unnatural to you?

Can you describe some features of farming you would call natural and non/unnatural?

***Cows***

What are your earliest memories of a cow of any kind, real or fictitious?

What are the differences between that cow you remember and cows now?

How natural should a dairy cow’s life be

Where is the line?

Describe how we could make a dairy cow’s life more natural?

**We’re now near the end of the interview.**

We’ve talked about your images of a dairy farm, and how they relate to these scenarios. We’ve covered what grazing and welfare and comfort mean, a bit about the environment, and have also talked about naturalness.

Is there anything else about dairy cows and dairy farming we haven’t covered that you’d like to raise?

**To round this up, could you give me three things that you feel should happen on your ideal dairy farm?**
